# The INFLORESCENCE DEFICIENT IN ABSCISSION-LIKE6 Peptide Functions as a Positive Modulator of Leaf Senescence in *Arabidopsis thaliana*

**DOI:** 10.3389/fpls.2022.909378

**Published:** 2022-06-30

**Authors:** Cun Guo, Xiaoxu Li, Zenglin Zhang, Qi Wang, Zhenbiao Zhang, Lichao Wen, Cheng Liu, Zhichao Deng, Yumeng Chu, Tao Liu, Yongfeng Guo

**Affiliations:** ^1^Chinese Academy of Agricultural Sciences, Tobacco Research Institute, Qingdao, China; ^2^Graduate School of Chinese Academy of Agricultural Sciences, Beijing, China; ^3^Technology Center, China Tobacco Hunan Industrial Co., Ltd., Changsha, China; ^4^QuJing Tobacco Company, Qujing, China

**Keywords:** leaf senescence, IDL6, plant peptide, *Arabidopsis*, transcriptome analysis, phytohormone

## Abstract

Leaf senescence is a highly coordinated process and has a significant impact on agriculture. Plant peptides are known to act as important cell-to-cell communication signals that are involved in multiple biological processes such as development and stress responses. However, very limited number of peptides has been reported to be associated with leaf senescence. Here, we report the characterization of the INFLORESCENCE DEFICIENT IN ABSCISSION-LIKE6 (IDL6) peptide as a regulator of leaf senescence. The expression of *IDL6* was up-regulated in senescing leaves. Exogenous application of synthetic IDL6 peptides accelerated the process of leaf senescence. The *idl6* mutant plants showed delayed natural leaf senescence as well as senescence included by darkness, indicating a regulatory role of IDL6 peptides in leaf senescence. The role of IDL6 as a positive regulator of leaf senescence was further supported by the results of overexpression analysis and complementation test. Transcriptome analysis revealed differential expression of phytohormone-responsive genes in *idl6* mutant plants. Further analysis indicated that altered expression of IDL6 led to changes in leaf senescence phenotypes induced by ABA and ethylene treatments. The results from this study suggest that the IDL6 peptide positively regulates leaf senescence in *Arabidopsis thaliana*.

## Introduction

As a vital part in plants’ life cycle, leaf senescence is a type of post-mitotic senescence which involves a strictly programmed cell death process (Gan, 2003). During leaf senescence, cellular organelles and macromolecules are degraded and nutrients are remobilized to reproductive organs and new tissues (Li et al., 2017; Guo et al., 2021). In agricultural applications, artificially accelerating or delaying leaf senescence in crop plants could achieve higher yields and better quality (Gan, 2014; Guo and Gan, 2014; Woo et al., 2019). During the past two decades, a large number of stay-green loci and senescence regulators have been identified from model plant systems which can be potentially used in manipulating leaf senescence for crop improvement (Vadez et al., 2011; Guo et al., 2015; Del Pozo et al., 2016).

Leaf senescence is an extremely complicated process and can be triggered by diverse factors including internal and external factors (Guo and Gan, 2005). The former includes phytohormones and reproduction growth (Lim et al., 2007). For instance, abscisic acid (ABA; He et al., 2005), ethylene (Iqbal et al., 2017), Jasmonate (JA; Hu et al., 2017) and salicylic acid (SA; Abreu and Munné-Bosch, 2008) accelerate leaf senescence, while cytokinin (CK; Hwang et al., 2012), auxin (IAA; Lim et al., 2010) and gibberellins (GA; Rodrigues et al., 2012) are negative regulators of senescence. External senescence-regulating factors are complex and generally associated with biotic/abiotic stresses, such as darkness, salinity, drought, extreme temperature and pathogen infection. Interestingly, some genes involved in biotic/abiotic stresses have also been reported to regulate leaf senescence. *WRKY55* was reported to be involved in both leaf senescence and disease resistance by regulating the accumulation of reactive oxygen species (ROS) and SA (Wang et al., 2020). In rice, *ONAC106* can be induced by salt stress, and functions as a negative regulator of leaf senescence (Sakuraba et al., 2015).

As an important part of cell-to-cell interaction in higher plants, peptide signals have been characterized to be involved in various aspects of plants’ life cycle including meristem organization (Guo et al., 2015), self-incompatibility (Liu et al., 2021), reproduction (Kim et al., 2021), organ abscission (Jinn et al., 2000), root growth (Huang et al., 2006), stress responses (Yamaguchi et al., 2010), hormone signaling (Estornell et al., 2013), nodule development (Mohd-Radzman et al., 2016) and RNA metabolism (Matsubayashi and Sakagami, 2006; Zhang et al., 2020). INFLORESCENCE DEFICIENT IN ABSCISSION (IDA) and IDA-Like (IDL) peptides are a small subgroup of plant peptides with 9 members in *Arabidopsis*, which possess an N-terminal signal peptide and a C-terminal extended PIP (EPIP) domain (Stenvik et al., 2008a,b). Some IDL peptides have been characterized for their roles in cell separation and stress responses. The IDA peptide was identified to control floral organ abscission and lateral root emergence *via* interacting with its receptors HAESA (HAE) and HAESA-LIKE2 (Stenvik et al., 2008b; Kumpf et al., 2013; Liu et al., 2013). The IDA-HAE/HSL2 signaling module functions in activating the mitogen-activated protein (MAP) kinase cascades, which in turn regulate the expression of cell wall-modifying and hydrolytic enzymes (Kumpf et al., 2013; Meng et al., 2016). In addition, SOMATIC EMBRYOGENESIS RECEPTOR KINASE 1 (SERK1) has been shown to act as a co-receptor of the IDA peptide in regulating flower abscission (Santiago et al., 2016). The coding genes of IDL6 and IDL7 peptides were shown to be induced rapidly by various stresses and have been suggested to be negative modulators of stress-induced ROS signaling (Vie et al., 2015, 2017). A recent study showed that the IDL6-HAE/HSL2 signaling module functions in facilitating infection of *Pseudomonas syringae* pv. tomato (*Pst*) DC3000 by promoting pectin degradation in *Arabidopsis* leaves (Wang et al., 2017).

A recent study reported that the CLAVATA3/ESR-RELATED 14 (CLE14) peptide serves as a senescence-regulating signal in *Arabidopsis* (Zhang et al., 2021), raising the possibility of more peptide signals involved in leaf senescence. Here we describe the characterization of the IDL6 peptide in regulating leaf senescence of *Arabidopsis*. The expression of *IDL6* was detected to be up-regulated in senescing leaves. The loss-of-function *idl6* mutant displayed a delayed senescence phenotype, and this phenotype could be rescued by the *IDL6* gene. Overexpressing *IDL6* or exogenous application of synthetic IDL6 peptides accelerated leaf senescence. Transcriptome analysis showed differential expression of phytohormones-responsive genes in *idl6* mutant plants. Further test of ABA and ethylene-induced senescence on detached leaves suggest that IDL6 might function *via* affecting ABA and ethylene signaling. Taken together, the results from this study indicate that IDL6 is a positive modulator of leaf senescence in *Arabidopsis*.

## Materials and Methods

### Plant Materials and Growth Conditions

*Arabidopsis* Columbia ecotype (Col-0) was used as the wild type in this study. The *idl6* mutant (SALK_074245) was obtained from the *Arabidopsis* Biological Resource Center (ABRC) and genotyped *via* PCR. The transcript abundance of the *IDL6* gene in *idl6* and Col-0 plants was detected by quantitative real-time PCR (qRT-PCR).

*Arabidopsis* seeds were surface sterilized with 70% (v/v) ethanol for 5 min and spread evenly in half-strength Murashige and Skoog (1/2 MS) media. Then the media were cultivated at 23°C with continuous light after being placed in a 4°C refrigerator for 2 days. Two weeks later, seedlings were transplanted to soil mixture, and kept in a growth chamber at 23°C with continuous light (Li et al., 2021; Zhang et al., 2021). Leaves at different development stages were selected for gene expression analysis. The fifth and sixth rosette leaves at different developmental stages were collected to explore the expression pattern of *IDL6* (YL, young leaves, 4-week-old; NS, non-senescence, 4.5-week-old; ES, early senescence, 5.5-week-old, LS, late senescence, 6.5-week-old). To this end, leaf samples of three biological replicates were collected and frozen in liquid nitrogen for RNA extraction.

### Generation of Constructs and Transgenic Plants

For overexpression analysis, *IDL6* (AT5G05300) CDS was PCR amplified from cDNA of Col-0 leaves. The PCR product was purified and inserted into the enzyme digested pCHF3 vector with Sac I by Infusion (Clontech, Beijing, China). The promoter sequence of *IDL6* was amplified from genomic DNA of Col-0 leaves and the PCR product was purified and inserted into the enzyme digested pBI121a vector (modified from pBI121) with Sac I by Infusion. Similarly, the promoter plus CDS fragment of *IDL6* was cloned into pZP211 for complementation test. All constructs were confirmed by Sanger sequencing and transformed into *Agrobacterium* competent cells (GV3101), which were used to transform *Arabidopsis* by *Agrobacterium*-mediated floral dip method (Zhang et al., 2006). The positive transgenic plants were screened on 1/2 MS medium containing 50 mg/l kanamycin and T3 homozygous lines were used for further study.

### RNA Extraction and qRT-PCR

Total RNAs from each sample were extracted by using the Ultrapure RNA Kit (cwbiotech, Beijing, China). Reverse transcriptions were performed using the Evo M-MLV Mix Kit with gDNA Clean for qPCR (Accurate Biotechnology, Changsha, China). qRT-PCR was performed using a Roche LightCycler 480 Real-Time PCR instrument with SYBR^®^ Green Premix Pro Taq HS qPCR Kit (Accurate Biotechnology, Changsha, China). *ACT2* was used as an internal control and all experimental data were obtained with three technical repetitions. The resulted Data were analyzed *via* the 2^–ΔΔCt^ method (Livak and Schmittgen, 2001). All primers used in this study are listed in Supplementary Table 1.

### Determination of Fv/Fm, Chlorophyll Content and Ion Leakage

The chlorophyll fluorescence Fv/Fm of individual leaves was determined using the IMAGING-PAM Mseries Chlorophyll Fluorescence System (LI-6400-40 LCF, Walz, Effeltrich, Germany) according to the manufacturer’s instructions (Rossel et al., 2006). For determination of chlorophyll content, 100% methanol was used in dissolving chlorophyll from leaves. After chlorophyll was completely released, absorbance at 666 and 653 nm was obtained with a spectrophotometer (ClarioSTAR, BMG LABTECH, Offenburg, Germany), chlorophyll content was calculated as previously described (Lightenthaler, 1987). For ion leakage measurement, leaves were immersed in deionized distilled water, shaken at 25°C for 30 min, and the beginning conductivity was measured using a digital conductivity meter (Thermo Fisher Scientific Traceable, Hampton, NH, United States of America). The samples were then boiled for 15 min and then the second conductivity was measured. The percentage of the first measurement over the second measurement was used as the membrane leakage indicator (Zhao et al., 2018).

### Peptide Synthesis and Detached Leaf Senescence Assay

The IDL6 peptide EPIP-domain (Supplementary Figure 1) sequence FGSLVLNALPKGSVPASGPSKRIN was synthesized by Genscript (Nanjing, China) at the purity of 95%. For detached leaf senescence assay, the seventh and eighth leaves of 8-week-old wild-type plants were excised and incubated in plates containing 1 μM IDL6 peptides. Leaf senescence phenotypes were recorded and chlorophyll contents were measured.

### Dark- and Phytohormone-Induced Leaf Senescence

All plants were grown on soil in a growth chamber under continuous light at 23°C. For dark-induced leaf senescence analysis, the fifth, sixth and seventh leaves of 4.5-week-old wild-type, *idl6* mutant, overexpression and rescued lines were excised and placed onto moistened filter paper inside foil-wrapped petri dishes as described previously (Li et al., 2016). Pictures were taken 0 d, 3 d, and 5 d after treatments. For hormone-induced senescence treatments, the seventh, eighth and nineth leaves of 6-week-old plants were incubated in liquid 1/2 MS media with or without 10 μM ABA or 50 μM ethephon, respectively, as described previously (Li et al., 2021). Pictures were taken at 0 d, 1 d, 3 d, and 5 d after treatments. For all measurements, three biological replicates were performed.

### RNA-Sequencing Analysis

The sixth and seventh leaves of 4-week-old Col-0 and *idl6* plants were collected and frozen in liquid nitrogen immediately. Each plant sample was represented by three biological replicates. The samples were entrusted to Shanghai OE Biotech for RNA-Seq. The quality of the sequencing data was scrutinized in terms of total raw reads, total clean reads, Q20 percentage, and GC percentage. DEGs were filtered using the following criteria: |Log2 (fold change)| > 2.0, *p* < 0.05. KEGG enrichment analysis was based on the path entries with the number of corresponding differential genes greater than 2, and sorted according to the corresponding -log10 *p*-value. Raw RNA-seq reads are available at the National Center for Biotechnology Information (BioProject ID: PRJNA821657).

### Statistical Analysis

All data analyses in this study were performed based on at least three biological replicates. Statistically significant differences were determined using Student’s *t*-test (*p < 0.05, **p < 0.01, and ***p < 0.001). Values in graphs are the mean value ± SE of all replicates.

## Results

### *ILD6* Is Up-Regulated During Leaf Senescence

**Figure 1:**
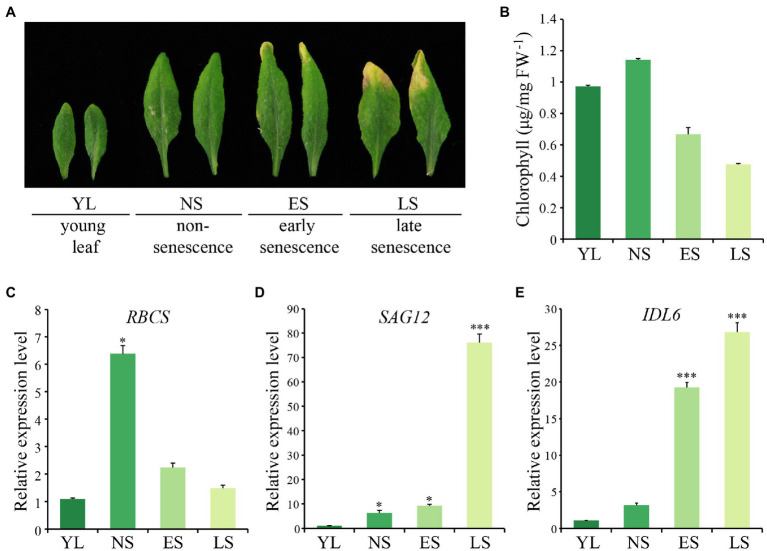
The expression pattern of *IDL6* during leaf senescence. **(A)** The *Arabidopsis* wild-type leaves at different developmental stages. YL: young leaf, NS: non-senescence leaf, ES: early senescence leaf, LS: late senescence leaf. **(B)** The chlorophyll contents of leaves at different developmental stages. **(C–E)** The transcript abundance of *RBCS*, *SAG12* and *IDL6* during leaf senescence. The data are means ± SD of three biological repeats. Significant difference compared with the YL was determined by Student’s *t*-test (*p < 0.05 and ***p < 0.001).

The 5th and 6th rosette leaves of *Arabidopsis* (Col-0) plants were collected to explore the expression pattern of *IDL6* at four different developmental stages, including young leaf (YL), non-senescence leaf (NS), early senescence leaf (ES) and late senescence leaf (LS; Figure 1A). Chlorophyll contents decreased from NS to LS stage, indicating progression of leaf senescence (Figure 1B). As expected (Gan and Amasino, 1995; Zhang et al., 2012; Guo et al., 2021), the rubisco small subunit encoding gene *RBCS* was down-regulated during leaf senescence (Figure 1C), while the senescence marker gene *SAG12* was up-regulated from NS stage to LS stage, and was highly expressed at the late senescence stage (Figure 1D). The *IDL6* transcripts were detected to be highly expressed in leaves at both early and late senescence stages (Figure 1E).

**Figure 2:**
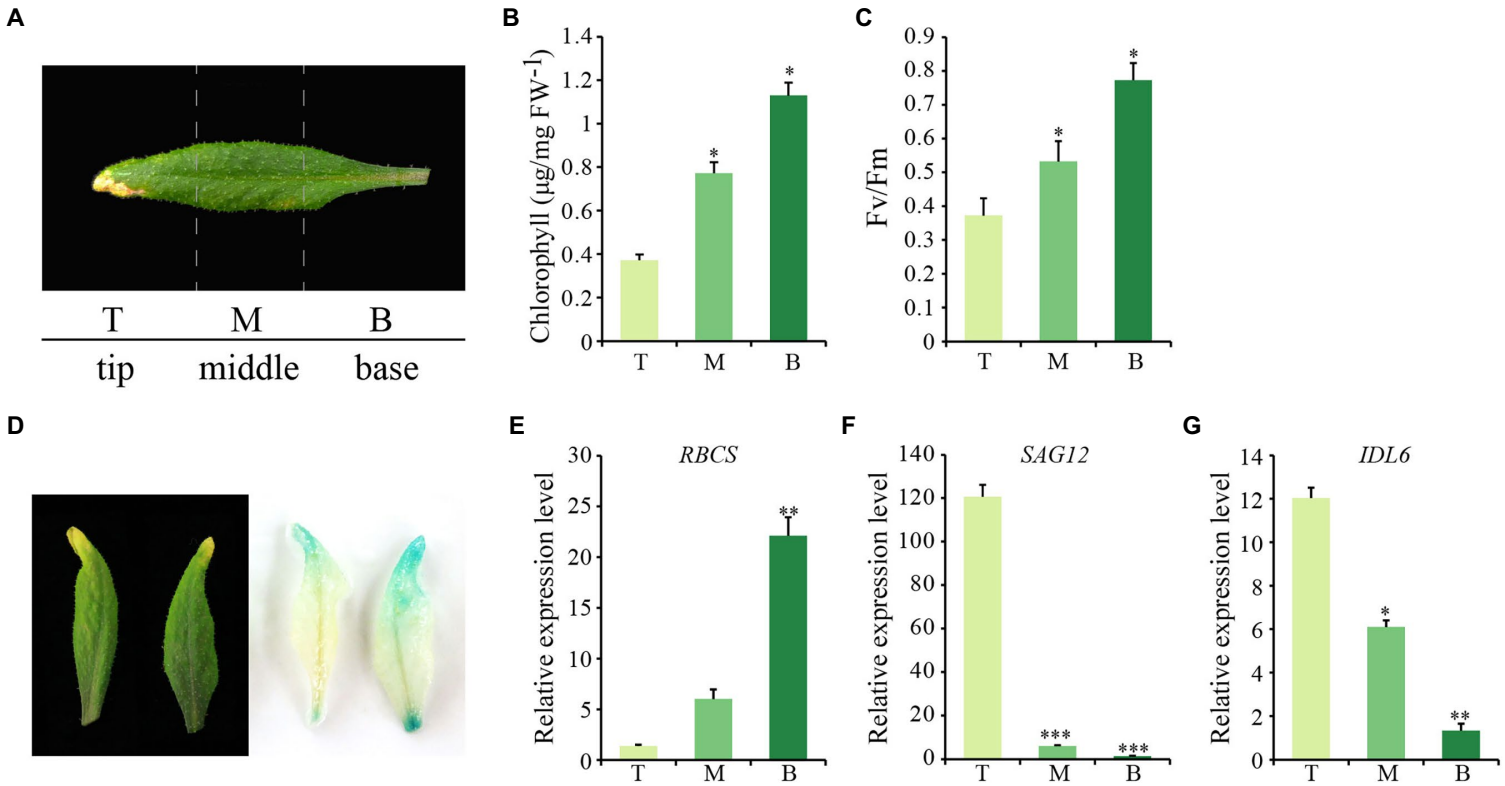
The expression of *IDL6* in different parts of a senescing leaf. **(A)** Representative image of three sections of *Arabidopsis* wild-type leaves. T: leaf tip, M: middle part, **B**: leaf base. Chlorophyll contents **(B)** and photosynthetic rates **(C)** in different leaf sections. **(D)**: GUS staining on 7th and 8th rosette leaves from 8-week-old *ProIDL6*::*GUS* transgenic line. **(E-G)** Transcript abundance of *RBCS*, *SAG12* and *IDL6* in different leaf sections. The data are means ± SD of three biological repeats. Significant differences compared with the tip part were determined by Student’s *t*-test (*p < 0.05, **p < 0.01, and ***p < 0.001).

Considering that natural senescence of a single leaf proceeds from the tip to the base part, an early senescence leaf from the 6th position of 8-week-old wild-type *Arabidopsis* was collected for analysis. From this leaf, three sections were isolated, including the tip, the middle and the base (Figure 2A). As expected, chlorophyll contents and photosynthetic rates declined gradually from tip to base (Figures 2B,C). Additionally, the transcript abundance of *SAG12* and *RBCS* were correlated with the degree of leaf senescence, with the highest expression levels of *RBCS* and *SAG12* detected in the base and the tip part, respectively, (Figures 2E,F). *IDL6* gene was expressed similarly to *SAG12*, which increased continuously from the leaf base to the tip and was highly expressed in the senescent leaf tip (Figure 2G). Furthermore, the *IDL6* promoter was cloned in front of the *b-glucuronidase* (*GUS*) gene and used for transforming wild-type plants. When *ProIDL6::GUS* leaves at the early senescence stage were used for GUS staining, more GUS signals were detected at the tip of leaves. Notably, strong GUS activity was observed at the base part of mechanically damaged leaves, suggesting that *IDL6* was also induced by wounding (Figure 2D).

### Exogenous Application of Synthetic IDL6 Peptides Accelerates Leaf Senescence

**Figure 3:**
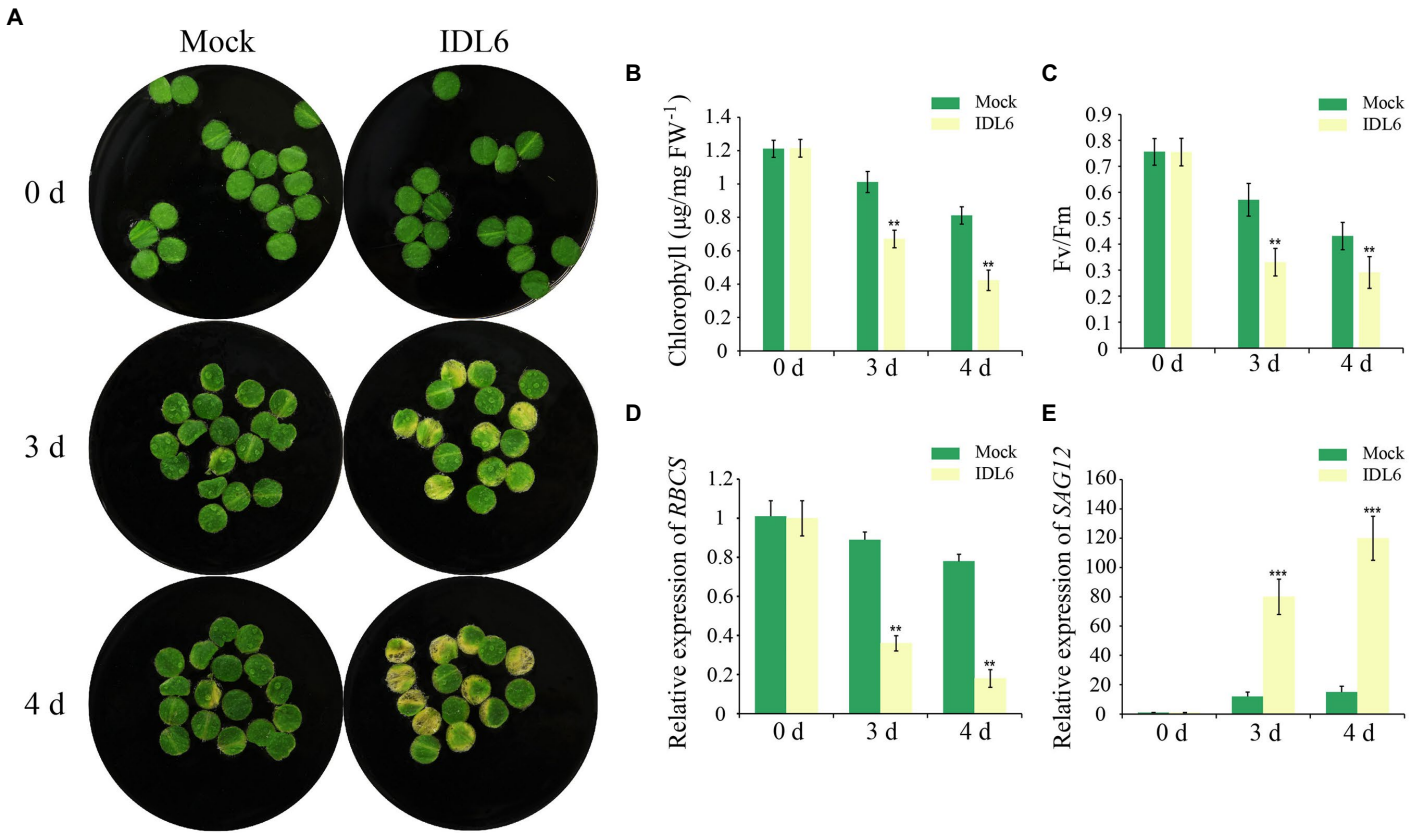
The senescence phenotypes of detached leaves treated with synthetic IDL6 peptides. **(A)** The detached leaves from wild type of *Arabidopsis* exhibited early senescence under IDL6 EPIP peptide treatments. Leaves were kept in a growth chamber at 23°C with continuous light. Changes in chlorophyll content **(B)** and photosynthetic rate **(C)** in the treated leaves. The relative expression of *RBCS* **(A)** and *SAG12* **(E)** in leaves from each group. Error bars indicate the SE (*n* > 10). Significant difference compared with the Mock was determined by Student’s *t*-test (**p < 0.01 and ***p < 0.001).

As a special group of plant hormones, peptide ligands could be artificially synthesized to explore their function. In previous studies, the EPIP-domain (extended PIP) of AtIDA and MiIDL1 were synthesized in determining their roles in floral organ shedding (Stenvik et al., 2008b; Kim et al., 2018). The EPIP sequence of IDL6 peptide was confirmed by sequence alignment according to previous studies (Butenko et al., 2003; Stenvik et al., 2008b) and was artificially synthesized for treating detached leaves (Supplementary Figure 1). Leaf disks of the seventh and eighth leaves from 8-week-old wild-type plants were treated with 1 μM IDL6 EPIP peptides. Three and 4 days later, an early senescence phenotype can be observed on the leaf disks treated with IDL6 EPIP peptides compared to mock treatments (Figure 3A). In consistent with visible phenotypes, significant decline of chlorophyll content and Fv/Fm was detected in leaves treated with IDL6 EPIP peptides in comparison with mock treatments (Figures 3B,C). Besides, the *RBCS* expression level of mock treatments was higher than peptide treatments, while, the *SAG12* expression level of mock treatments was lower than peptide treatments (Figures 3D,E). This result suggests that the IDL6 EPIP peptide functions in accelerating leaf senescence.

### Loss of *IDL6* Function Delays Leaf Senescence

**Figure 4:**
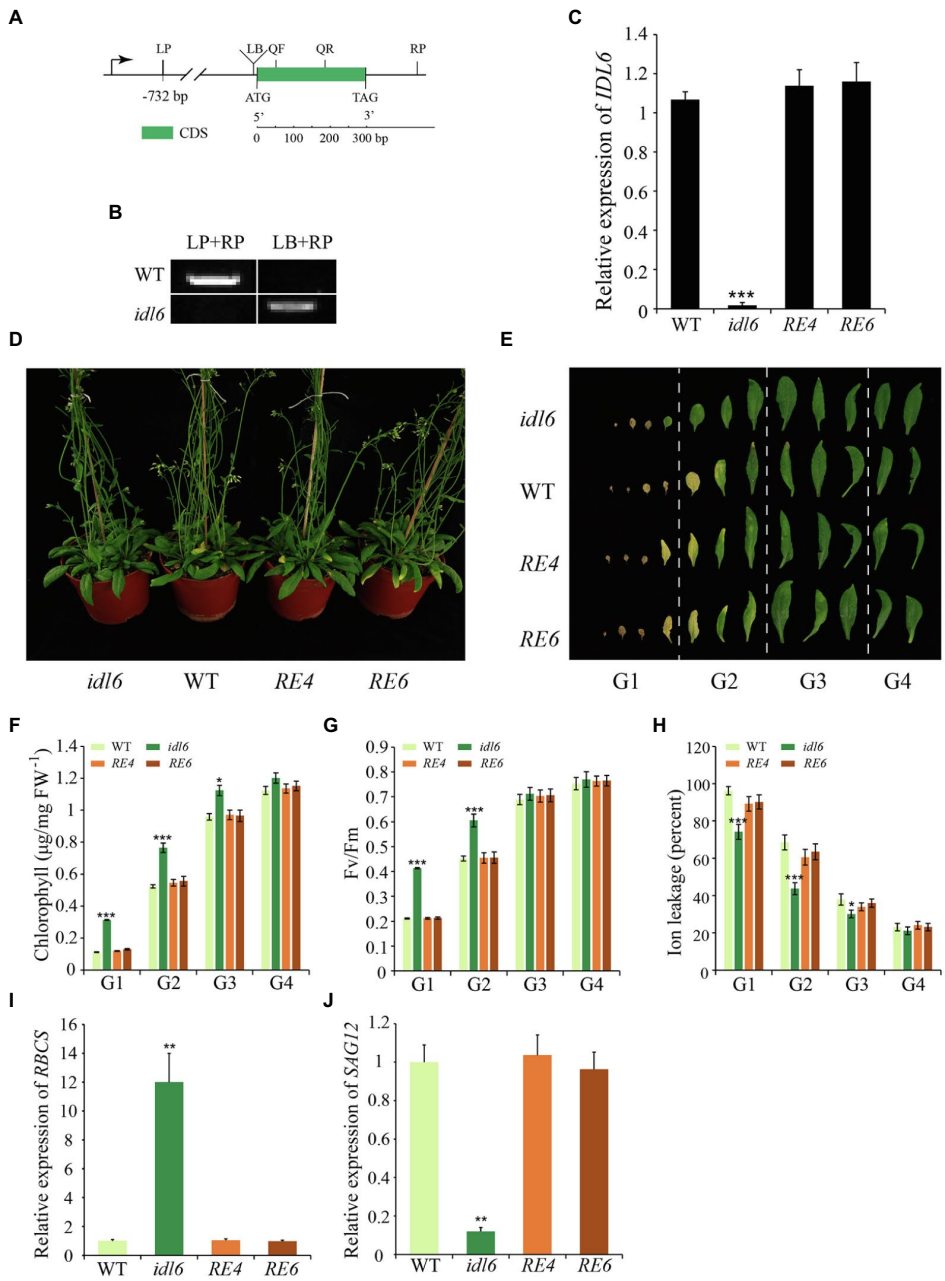
Leaf senescence phenotypes of *IDL6* loss-of-function plants. **(A)** The gene structure of the *IDL6* gene and location of the T-DNA insertion. **(B)** Homozygous mutant plants were identified by PCR. LP: left primer, RP: right primer, LB: T-DNA left border primer. **(C)** Quantification of *IDL6* transcripts in wild-type, *idl6* mutant and *IDL6* rescue lines. **(D)** Leaf senescence phenotypes of 8-week-old wild-type, *idl6* mutant and *IDL6* rescue lines (*RE4* and *RE6*). **(E)** Phenotypes of 12 detached rosette leaves from wild-type, *idl6* mutant and *IDL6* rescue lines. Quantification of chlorophyll contents **(F)**, photosynthetic rates **(G)** and ion leakage **(H)** in leaves from each group. The relative expression of *RBCS* **(I)** and *SAG12* **(J)** of group 2 leaves from each line. The data are means ± SD of three biological repeats. Significant differences (*p < 0.05, **p < 0.01, and ***p < 0.001) compared with the wild type in each group were determined by Student’s *t*-test.

To confirm the role of IDL6 in regulating of leaf senescence, a mutant line SALK_074245 (*idl6*) with a T-DNA insertion before the start codon of the *IDL6* gene was obtained (Figure 4A). PCR and Sanger sequencing were performed to identify homozygous *idl6* mutant plants. *IDL6* transcript was not detected in the late senescence leaf of *idl6* plants, suggesting that is *idl6* a null mutant (Figures 4B,C). Two rescue lines were obtained by transforming the *IDL6* gene back to *idl6* mutant plants (Figure 4C; Supplementary Figure 2).

No obvious phenotypic changes were found between the *idl6* mutant line, wild-type and rescue lines at early developmental stages. At alter stage, when 8-week-old plants were compared, the loss-of-function *idl6* mutant displayed a significant delay in leaf senescence. The delayed senescence phenotype of *idl6* plants was rescued to wild type in plants of the rescue lines (Figures 4D,E). To further characterize the senescence phenotypes of the *idl6* mutant, the 12 rosette leaves from plants of different genotypes were divided into four groups to collect physiological data (Figure 4E). As a result, the highest Fv/Fm ratio and chlorophyll contents were found in leaves of each group from the *idl6* mutant (Figures 4F,G). Ion leakage is an important plasma membrane integrity indicator, and is considered one of the most important indicators of leaf senescence (Feller and Fischer, 1994). The leaves of the *idl6* mutant showed significantly lower ion leakage rates compared with the wild-type and rescue lines (Figure 4H). As expected, the *RBCS* expression level of *idl6* plants was higher than that of the wild-type and complementary lines, while the *SAG12* expression level of the *idl6* mutant was lower than wild-type and complementary lines (Figures 4I,J).

### Overexpression of *IDL6* Accelerates Leaf Senescence

**Figure 5:**
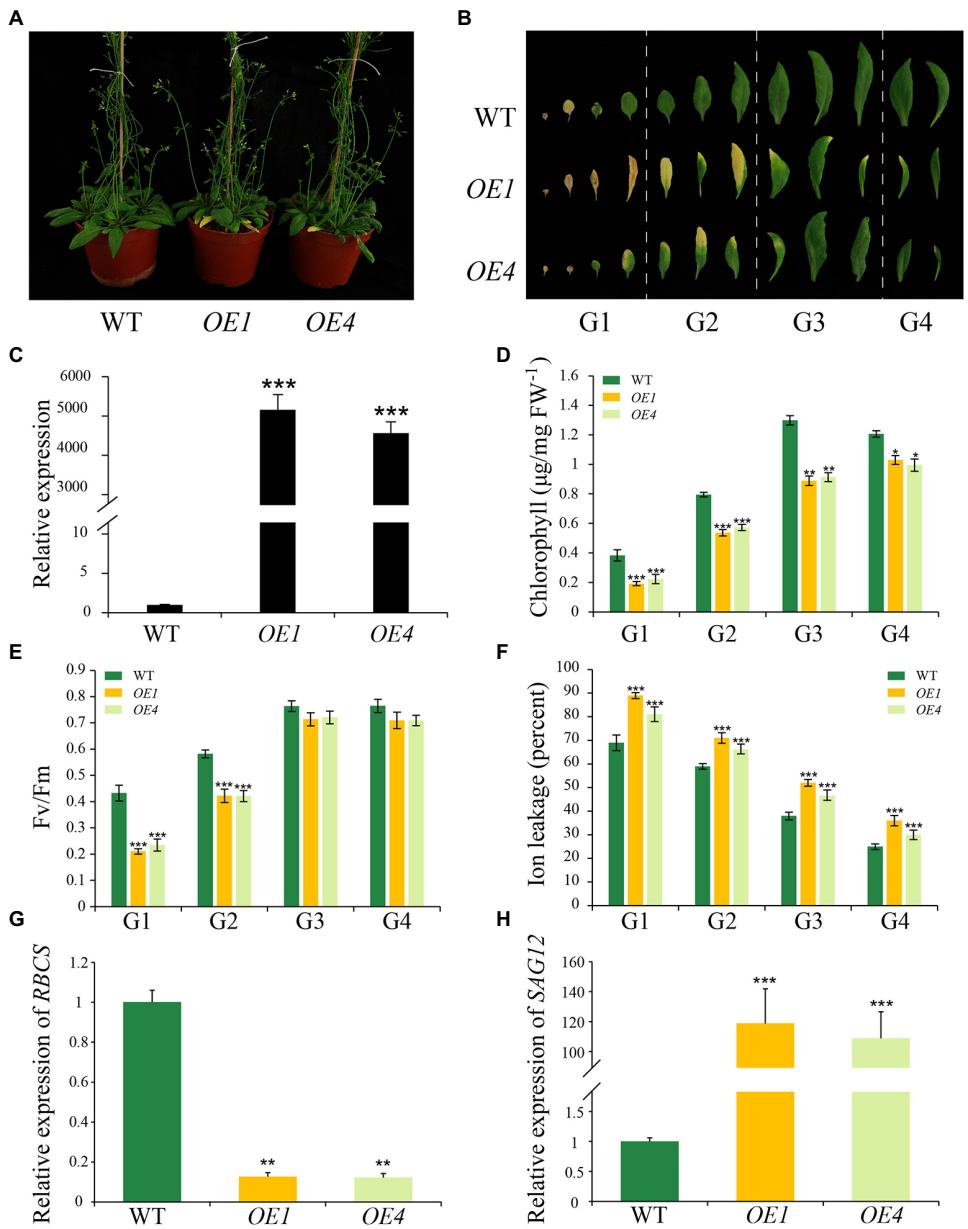
Leaf senescence phenotypes of *IDL6* overexpression plants. **(A)** Leaf senescence phenotypes of 6-week-old wild-type and *IDL6* overexpression lines (*OE1* and *OE4*). **(B)** Phenotypes of 12 detached rosette leaves from wild-type and *IDL6* overexpression lines. **(C)** Relative expression of the *IDL6* gene in wild-type and *IDL6* overexpression lines. Quantification of chlorophyll contents **(D)**, photosynthetic rates **(E)** and ion leakage **(F)** in leaves from 4 groups of rosette leaves. The relative expression of *RBCS* **(G)** and *SAG12* **(H)** of group 2 leaves from each line. The data are means ± SD of three biological repeats. Significant differences (*p < 0.05, **p < 0.01, and ***p < 0.001) compared with the wild type in each group were determined by Student’s *t*-test.

To further explore the function of IDL6 in leaf senescence, the *IDL6* gene was overexpressed using the 35S promoter. Two overexpression lines (namely *OE-1* and *OE-4*) were selected to for further analysis. The results showed that leaves of these overexpression lines exhibit morphologies of smaller size, which is consistent with what Wang et al. (2017) reported. The IDL6-OE lines displayed an early leaf senescence phenotype compared with wild type (Figures 5A–C). The chlorophyll contents and Fv/Fm values in the overexpression lines were significantly lower than that of wild-type plants (Figures 5D,E). The high ion leakage in the overexpression lines also suggested that the leaf senescence progression was accelerated with enhanced *IDL6* expression (Figure 5F). This was also demonstrated by the expression of *RBCS* and *SAG12* in wild-type and overexpressed plants (Figures 5G,H).

### Transcriptome Analysis Reveals the Importance of Hormone Signaling in IDL6-Mediated Leaf Senescence

Earlier studies suggested that 10–16% of all genes show differential expression during leaf senescence (Breeze et al., 2011; Woo et al., 2016). To explore gene expression changes caused by the *idl6* mutation, the 6th and 7th leaves with similar degree of yellowing from wild-type and *idl6* plants were collected to perform RNA-seq analysis.

**Figure 6:**
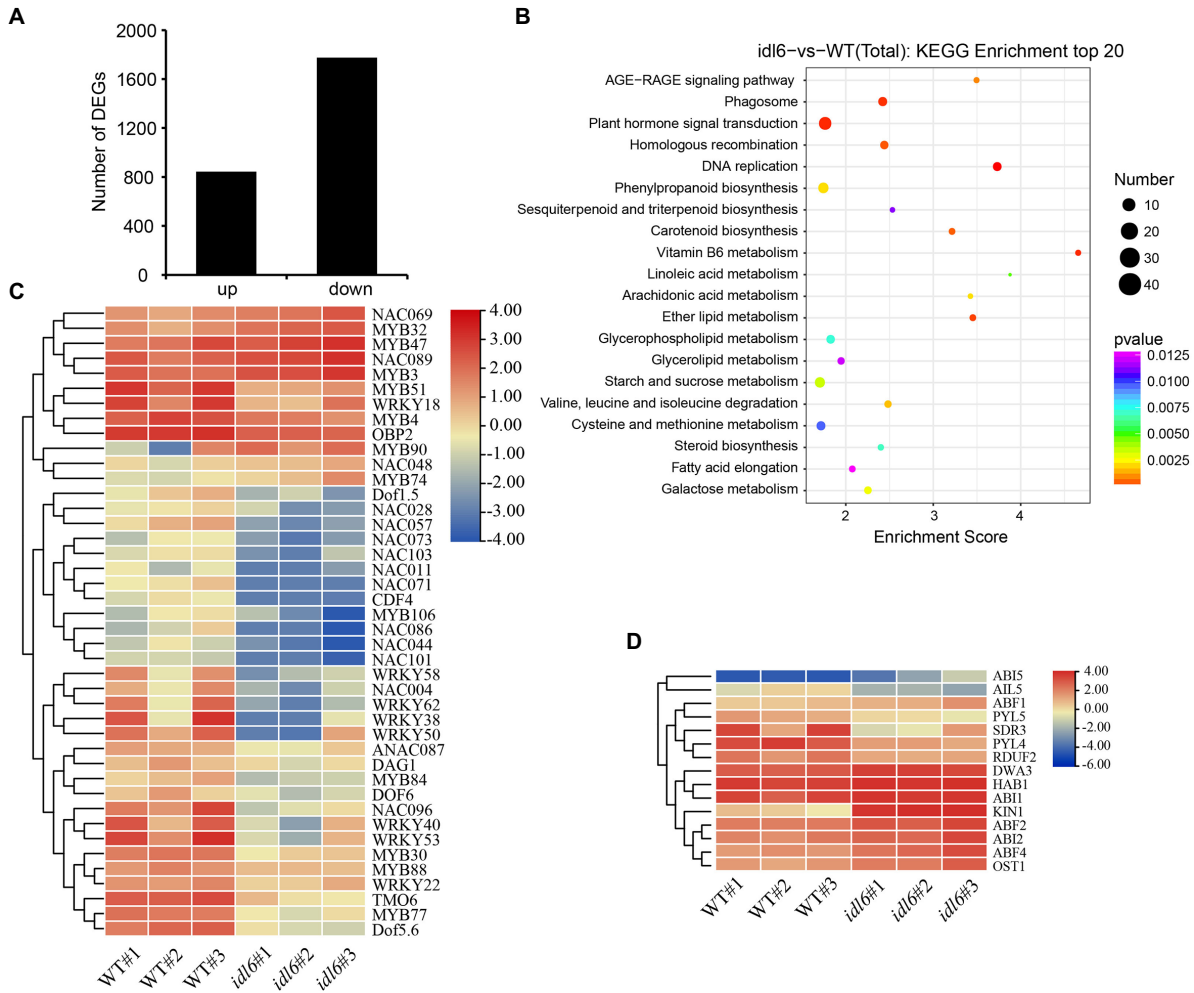
Genome-wide transcriptome analysis of *idl6* mutant plants. **(A)** The number of up-regulated and down-regulated genes in rosette leaves of *idl6* mutant vs. Col-0. **(B)** KEGG bubble diagram of the DEGs in rosette leaves of *idl6* vs. Col-0. Bubble size represents numbers of DEGs, and bubble color represents *p*-values. The enrichment score represents the significance degree of DEGs in a certain signaling pathway. **(C)** Heatmap showing leaf senescence-related transcription factors in *idl6* mutant leaves compared with Col-0. The log2 fold change scale is indicated on the right side of the heatmap. **(D)** The heatmap of ABA-related genes in *idl6* mutant leaves compared to Col-0. The log2 fold change scale is indicated on the right side of the heatmap.

The transcriptome analysis identified 2,618 differentially expressed genes (DEGs) in *idl6* mutant compared with wild type (Figure 6A; Supplementary Table 2). Interestingly, The WRKY transcription factor *WRKY53* (Miao et al., 2004) and Dof transcription factor *CDF4* (Xu et al., 2020), which have been reported to be positive regulators of leaf senescence, were significantly down-regulated in the *idl6* mutant (Figure 6C). Moreover, a number of stress responsive WRKY transcription factors, including *WRKY38* and *WRKY62* (Kim et al., 2008), were significantly down-regulated in *idl6* leaves (Figure 6C), Also, the expression levels of these genes were confirmed in wild-type, *idl6*, rescue and overexpression lines with qRT-PCR (Supplementary Figure 3).

When KEGG analyses of DEGs were performed to compare wild-type and the *idl6* mutant, more DEGs were enriched in plant hormone signaling pathways (Figure 6B). The ABA-INSENSITIVE1 (ABI1) protein phosphatase 2C and ABI2 were reported to be negative regulators of ABA signaling (Merlot et al., 2001). Both *ABI1* and *ABI2* were up-regulated in *idl6* mutant (Figure 6D). In addition, a number of genes related to ABA signal transduction, including *ABI5*, *PYL5*, *PYL4*, *OST1*, *ABF2* and *ABF4* (Finkelstein and Lynch, 2000; Mustilli et al., 2002; Kim et al., 2004; Santiago et al., 2009) showed differential expression in *idl6* plants (Figure 6D). These results suggest that IDL6 might be involved in hormonal-induced leaf senescence.

### IDL6 Might Be Involved in ABA- and Ethylene-Induced Leaf Senescence

**Figure 7:**
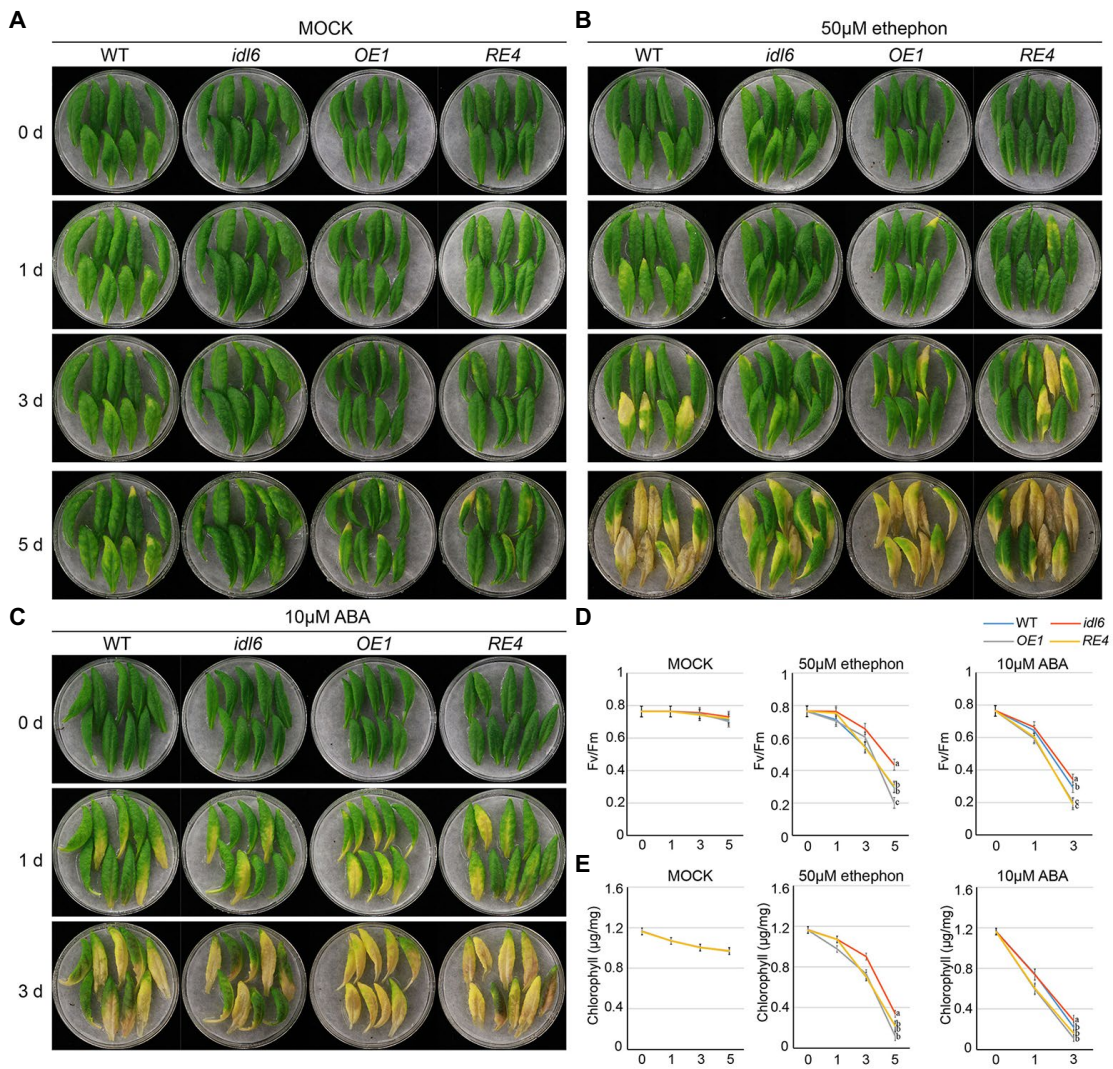
Senescence induced by ABA and ethylene on detached leaves of *Arabidopsis*. The 7th, 8th and 9th leaves of 6-week-old wild type, *idl6* mutant, *IDL6* OE plants and *idl6* rescue plants were collected and treated by mock **(A)**, 50 μM ethephon **(B)** and 10 μM ABA **(C)**. The images of leaves were taken before and 1 day, 3 days, and 5 days after treatments. **(D)** The Fv/Fm and **(E)** chlorophyll concentrations in hormone treated and untreated leaves. For all conditions, statistically significant had been performed by two-way ANOVA analysis. All treatments were performed for three times.

A growing body of evidence suggests that both ABA and ethylene are positive regulators of leaf senescence. In order to find out whether IDL6 is involved in leaf senescence induced by ABA or ethylene, the seventh, eighth and nineth leaves of wild-type, *idl6*, *IDL6-OE1* and *IDL6-RE4* plants were isolated and treated with 10 μm ABA or 50 μm ethylene. As expected, leaf senescence was accelerated by ABA and ethylene treatments. Significant leaf yellowing was observed on leaves from wild type 1 day after ABA treatments and 3 days after ethylene treatments. Leaves from the *idl6* mutant showed delayed while leaves from the *IDL6* overexpression line *OE1* showed accelerated senescence compared with wild type. The *IDL6* rescue-line showed the similar leaf senescence progress with wild type after both ABA and ethylene treatments (Figures 7A–C). Changes in photosynthetic rates and chlorophyll contents in the treated leaves were consistent with the leaf yellowing progression (Figures 7D,E). These results hint that IDL6 might be involved in ABA- and ethylene-induced leaf senescence.

### IDL6 Functions in Promoting Dark-Induced Senescence

**Figure 8:**
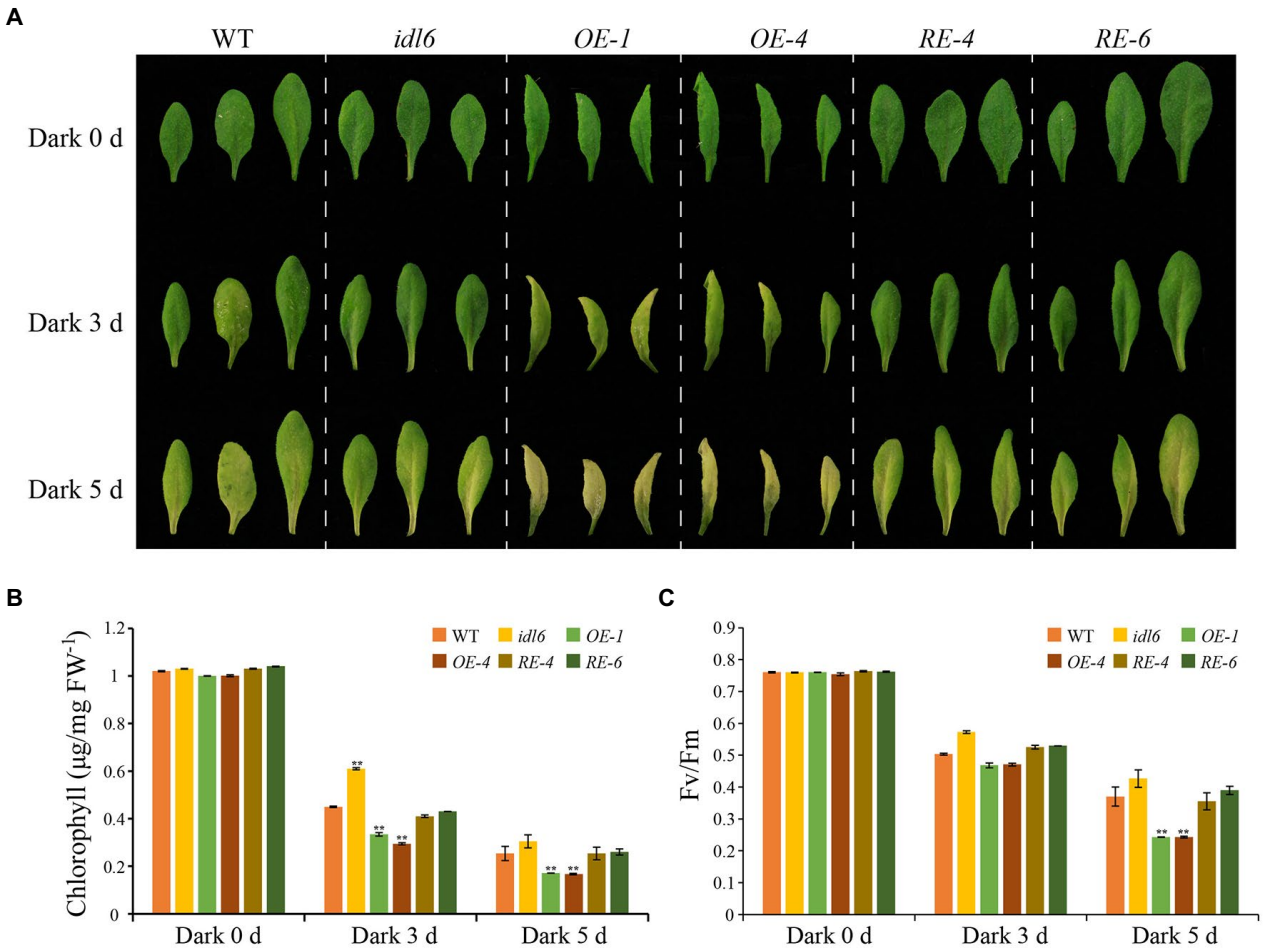
Dark-induced senescence in detached leaves with altered *IDL6* expression. **(A)** The senescence phenotype s of the 5th, 6th, and 7th leaves from 4.5-week-old plants under dark treatment. Chlorophyll contents **(B)** and photosynthetic efficiency **(C)** of each leaf were measured after dark treatment for 0, 3 and 5 days. Error bars showed the SE (*n* = 3). Significant differences (**p < 0.01) compared with the wild type in each group were determined by Student’s *t*-test. Three independent experiments were carried out with similar results.

To further explore the role of IDL6 peptides in leaf senescence, dark treatments were performed to induce leaf senescence. The 5th, 6th, and 7th leaves were excised from 4.5-week-old plants (WT, *idl6*, *OE1*, *OE4*, *RE4*, and *RE6*), placed on moistened filter papers and incubated under darkness to induce senescence. Three days later, the chlorophyll levels of all leaves from the *IDL6 OE* lines were significantly reduced, while relatively less chlorophyll breakdown occurred in the leaves of *idl6* mutant plants. After 5 days of dark treatment, the early senescence phenotype became more obvious on leaves from *IDL6 OE* lines (Figures 8A,B). Specifically, the chlorophyll contents in *IDL6 OE* lines were ~ 2.94, ~ 3.13, and 2.94 times lower than that of the wild-type, *idl6* mutant and *IDL6 RE* lines, respectively. The changes in Fv/Fm also showed a similar trend (Figures 8B,C). Notably, overexpression of *IDL6* could also affect *RBCS* and *SAG12* expression in dark-induced senescence (Supplementary Figure 4). These results suggest that IDL6 is a positive regulator of dark-induced leaf senescence.

## Discussion

Being stationary in nature, plants have evolved a series of sophisticated mechanisms for responding to unpredictable environmental stresses. Among them, cell-to-cell communication systems play a key role in growth and development. During the last decade, peptide ligands have emerged as vital mediators of cell-to-cell communications in plant growth, defense and stress responses in addition to the classical phytohormones (Matsubayashi and Sakagami, 2006). However, only one peptide, CLE14, has been functionally characterized and reported to be involved in leaf senescence (Zhang et al., 2021). In the current study, we found that *IDL6*, encoding a secreted peptide, was highly expressed in senescence leaves. Loss of *IDL6* function mutation delayed leaf senescence while *IDL6* overexpression and IDL6 peptide treatments caused precocious leaf senescence, supporting the role of IDL6 as a positive mediator of leaf senescence in *Arabidopsis*.

Comprising nine members (IDA and IDL1-8) in *Arabidopsis*, the IDL family members were detected to be expressed in floral organs, leaves, and roots. Among them, IDA has been shown to be important for flower abscission and lateral root emergence, while IDL6 and IDL7 have been reported to be negative regulators of genes associated with early responses to stresses (Vie et al., 2017). In a previous study, knockdown lines of *IDL6* showed increased resistance to *Pst* DC3000 in *Arabidopsis* (Wang et al., 2017). In this study, IDL6 peptides were found to function in promoting age-dependent leaf senescence and senescence induced by darkness, ABA and ethylene treatments. Cross-talks between plant senescence and stress responses have been well recognized in earlier studies (Guo and Gan, 2012; Guo et al., 2021). IDL6 could be induced rapidly by various biotic and abiotic stresses, such as cold, salt, UV, *P. syringae* (Vie et al., 2015). It might act as a signaling hub where different pathways interconnect with each other. Notably, peptides from the same family are often found to be involved in similar biological processes (Matsubayashi and Sakagami, 2006). It will be no surprise if some of the other IDL family peptides are also found to be involved in plant senescence.

Receptor-like kinases are indispensable sensors that contribute to intercellular communication, especially peptide ligand signaling (Diévart and Clark, 2003; Gish and Clark, 2011). At present, most receptors of peptides have been identified to be in the Leucine-rich repeat receptor-like kinases (LRR-RLK) family, such as RLK7, CEPR, BAM, and PEPR (Deyoung et al., 2006; Klauser et al., 2015; Zhou et al., 2022). As the receptors of the IDA peptide, HAE and HSL2 were reported to participate in flower abscission and lateral root emergence (Stenvik et al., 2008b; Kumpf et al., 2013). HAE and HSL2 also have been characterized as receptors of the IDL6 peptide in regulating plant disease resistance by activating cell wall synthesis genes (Wang et al., 2017). Peptide signals might be perceived by the same or different receptors when functioning in different biological processes (Zhang et al., 2021). Whether IDL6 functions through interacting with HAE and HSL2 in promoting leaf senescence remains to be elucidated.

The roles of phytohormones in leaf senescence have been well established (Guo and Gan, 2012). The transcriptome analysis in this study revealed multiple DEGs related to phytohormones in the *idl6* mutant (Figures 6B,D). Further study indicated that IDL6 functioned in leaf senescence induced by ABA and ethylene (Figure 7). How peptide signals including CLE14 and IDL6 interact with known senescence-regulating phytohormones and other senescence-regulating signals will be the next questions to be addressed in this field.

Leaf senescence as a complex and orderly controlled physiological process, requires hierarchical but also coordinated regulation by multiple transcription factors (Guo et al., 2004). The transcriptomic analyses of leaves from different developmental stages have identified numerous TFs differentially expressed during leaf senescence and more and more studies had characterized these TFs’ functions in leaf senescence (Guo, 2013). In this study, we found that *WRKY53* and *CDF4* were down-regulated in the *idl6* mutant (Figure 6C), while they showed higher expression in overexpression *IDL6* gene line (Supplemental Figure 3). These results indicated that transcription factors WRKY53 and CDF4, both have been characterized as positive regulators of senescence (Miao et al., 2004; Xu et al., 2020), might function downstream of IDL6 in regulating leaf senescence. The specific regulatory mechanisms underlying the interactions between IDL6 and senescence-regulating transcription factors remain to be further studied.

## Conclusion

In this study, we functionally characterized the IDL6 peptide, the encoding gene of which exhibited the highest expression level in naturally senescing leaves. Exogenous application of synthetic IDL6 EPIP peptides accelerated leaf senescence. Transgenic *Arabidopsis* plants with depleted or overexpressed *IDL6* had delayed or accelerated leaf senescence, respectively, indicating a positive role of IDL6 peptides in regulating leaf senescence. Furthermore, IDL6 peptides induced leaf senescence under darkness and hormonal treatments. Several senescence-associated transcription factors were significantly down-regulated in the *idl6* mutant, suggesting extensive cross talks between the IDL6 signal and known senescence-regulating pathways.

## Data Availability Statement

The datasets presented in this study can be found in online repositories. The names of the repository/repositories and accession number(s) can be found in the article/supplementary material.

## Author Contributions

CG and XL conducted the research and participated in drafting the manuscript. ZeZ, QW, ZhZ, LW, CL, ZD, YC, and TL assisted in data collection and analysis. YG conceived this research, designed the experiments, and drafted the manuscript. All authors contributed to the article and approved the submitted version.

## Funding

This research was funded by the Agricultural Science and Technology Innovation Program (ASTIP-TRIC02), Funds for Special Projects of the Central Government in Guidance of Local Science and Technology Development (21-1-1-1-zyyd-nsh), and National Natural Science Foundation of China (31571494).

## Conflict of Interest

XL was employed by China Tobacco Hunan Industrial Co., Ltd. CL was employed by QuJing Tobacco Company.

The remaining authors declare that the research was conducted in the absence of any commercial or financial relationships that could be construed as a potential conflict of interest.

## Publisher’s Note

All claims expressed in this article are solely those of the authors and do not necessarily represent those of their affiliated organizations, or those of the publisher, the editors and the reviewers. Any product that may be evaluated in this article, or claim that may be made by its manufacturer, is not guaranteed or endorsed by the publisher.

## Supplementary Material

The Supplementary Material for this article can be found online at: https://www.frontiersin.org/articles/10.3389/fpls.2022.909378/full#supplementary-material

Supplementary Figure 1 Sequence information of the IDL6 peptide.

Supplementary Figure 2 Growth of wild-type, *idl6* mutant and a complementary line on 1/2 MS medium containing Kanamycin.

Supplementary Figure 3 The relative expression of *WRKY53*, *CDF4*, *WRKY38*, and *WRKY62* in wild-type, *idl6* mutant, rescue and overexpression lines.

Supplementary Figure 4 The expression levels of *RBCS* and *SAG12* in wild-type and overexpression plants under dark treatments.

Supplementary Figure 5 The senescence phenotypes of detached leaves treated with 1 μM and 10 μM IDL6 peptides. **(A)** The detached leaves from wild type of *Arabidopsis* exhibited early senescence under IDL6 peptide treatments. Leaves were kept in a growth chamber at 23 °C with continuous light. Changes in chlorophyll content **(B)** and photosynthetic rate **(C)** in the treated leaves. Significant differences compared with the Mock were determined by Student’s *t*-test (^*^p < 0.05, ^**^p < 0.01).
