# Supplementary figures and images for: The INFLORESCENCE DEFICIENT IN ABSCISSION-LIKE6 Peptide Functions as a Positive Modulator of Leaf Senescence in Arabidopsis thaliana
Source: Front Plant Sci. 2022 Jun 30;13:909378. doi: 10.3389/fpls.2022.909378 (PMC9280484; doi:10.3389/fpls.2022.909378)

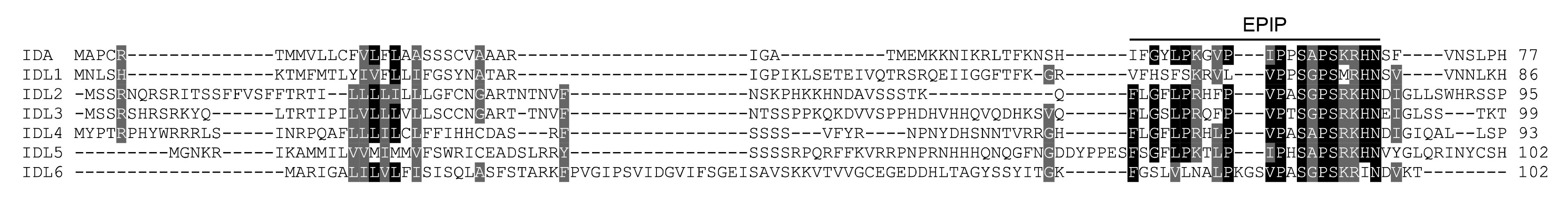

Supplement: Supplementary Figure 1 — Sequence information of the IDL6 peptide. [file Data_Sheet_1.ZIP › Supplementary Material/Supplementary Figure 1.jpg]

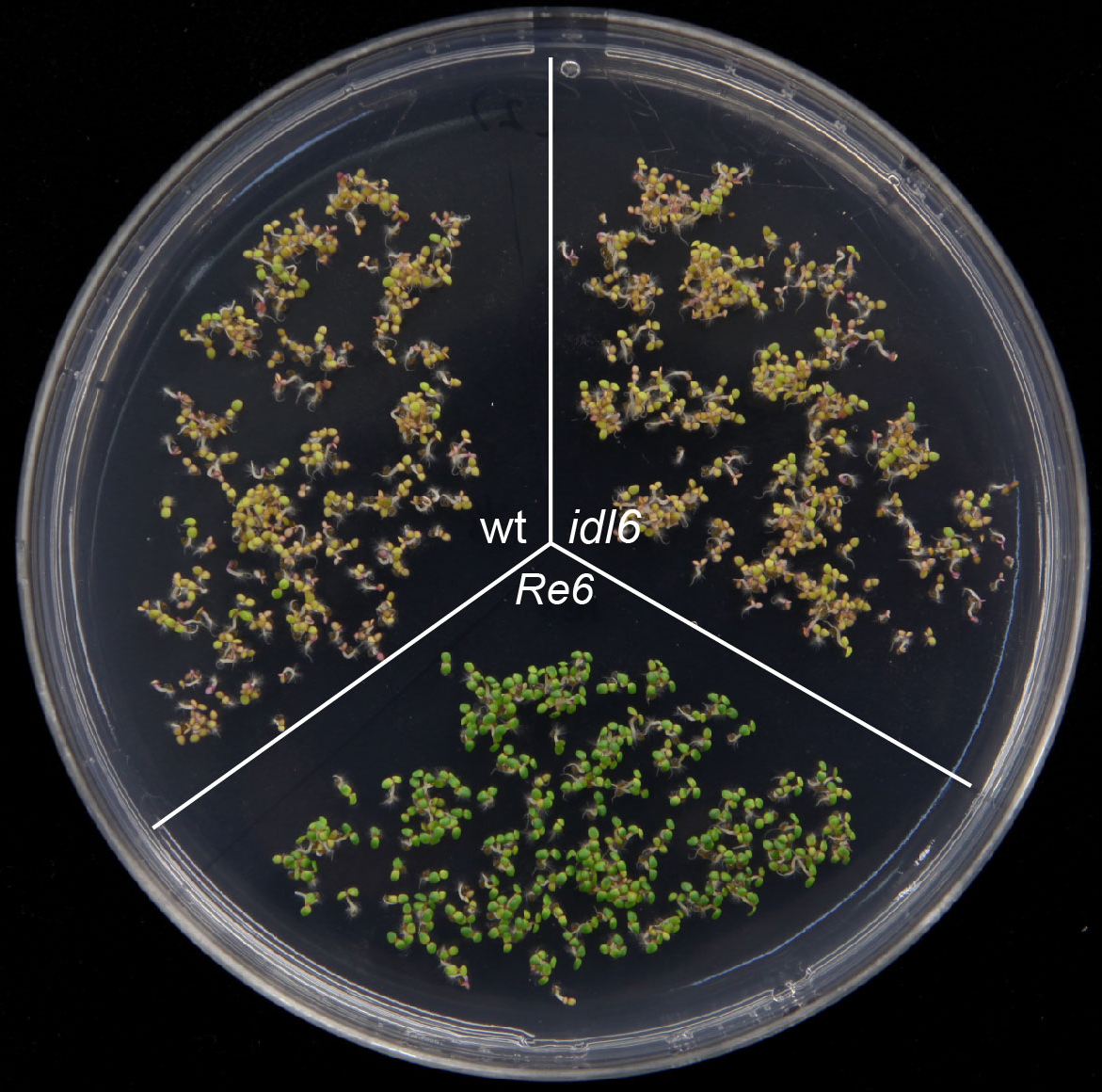

Supplement: Supplementary Figure 1 — Sequence information of the IDL6 peptide. [file Data_Sheet_1.ZIP › Supplementary Material/Supplementary Figure 2.JPG]

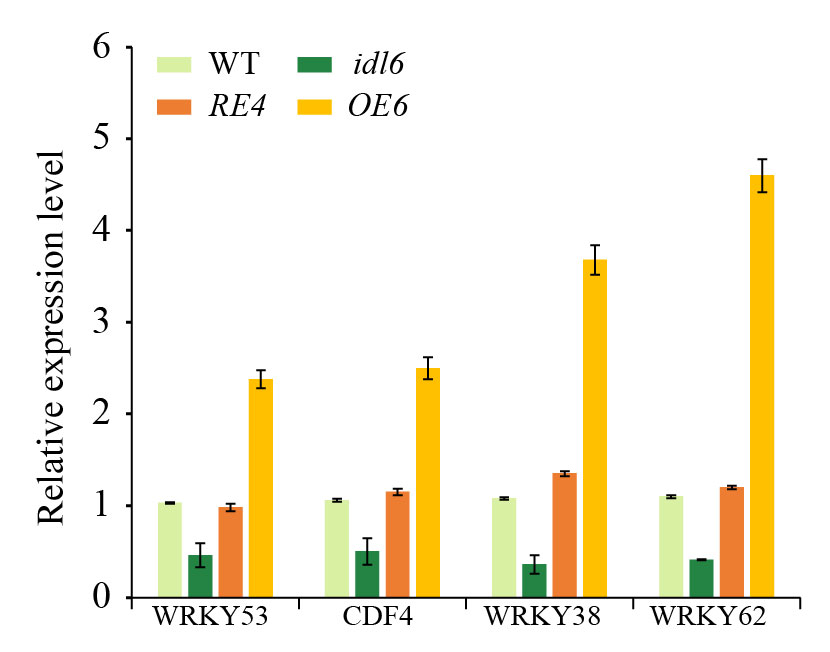

Supplement: Supplementary Figure 1 — Sequence information of the IDL6 peptide. [file Data_Sheet_1.ZIP › Supplementary Material/Supplementary Figure 3.jpg]

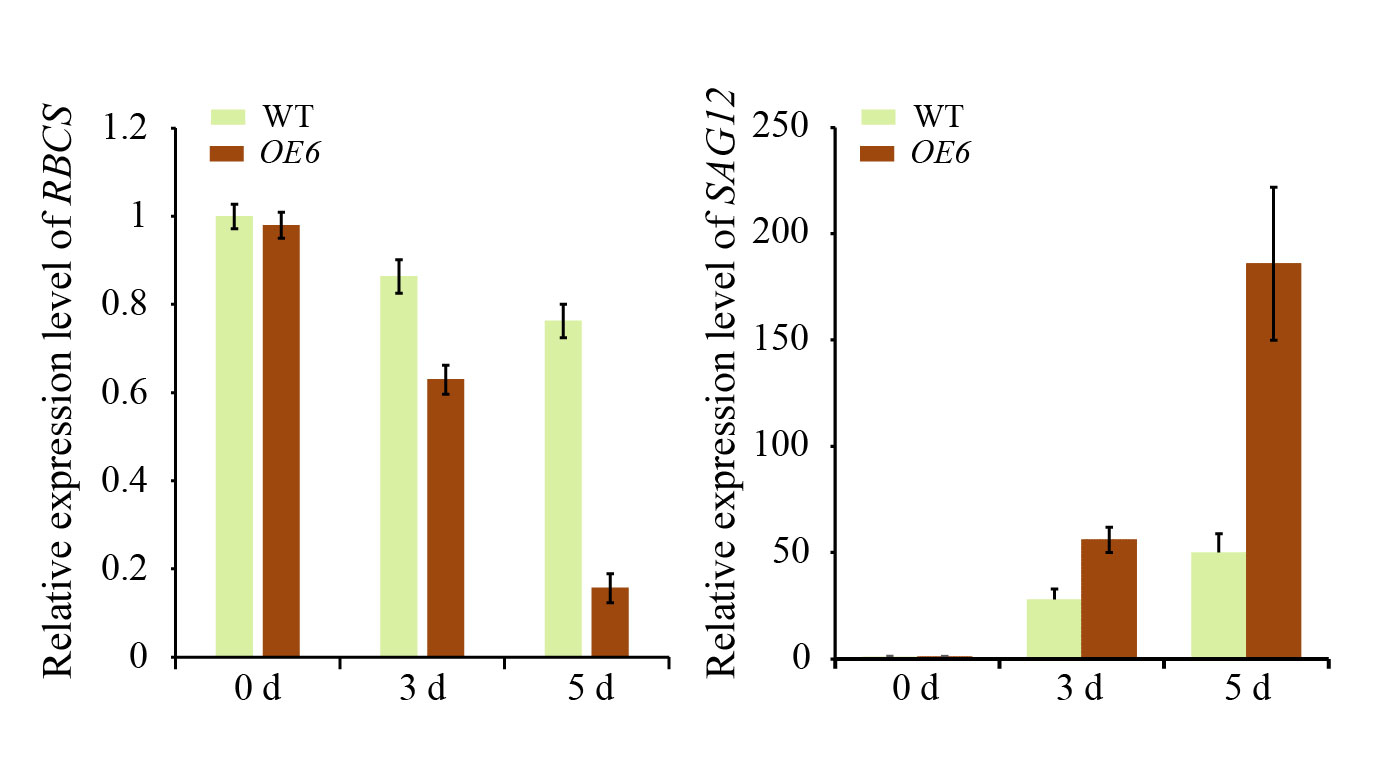

Supplement: Supplementary Figure 1 — Sequence information of the IDL6 peptide. [file Data_Sheet_1.ZIP › Supplementary Material/Supplementary Figure 4.jpg]

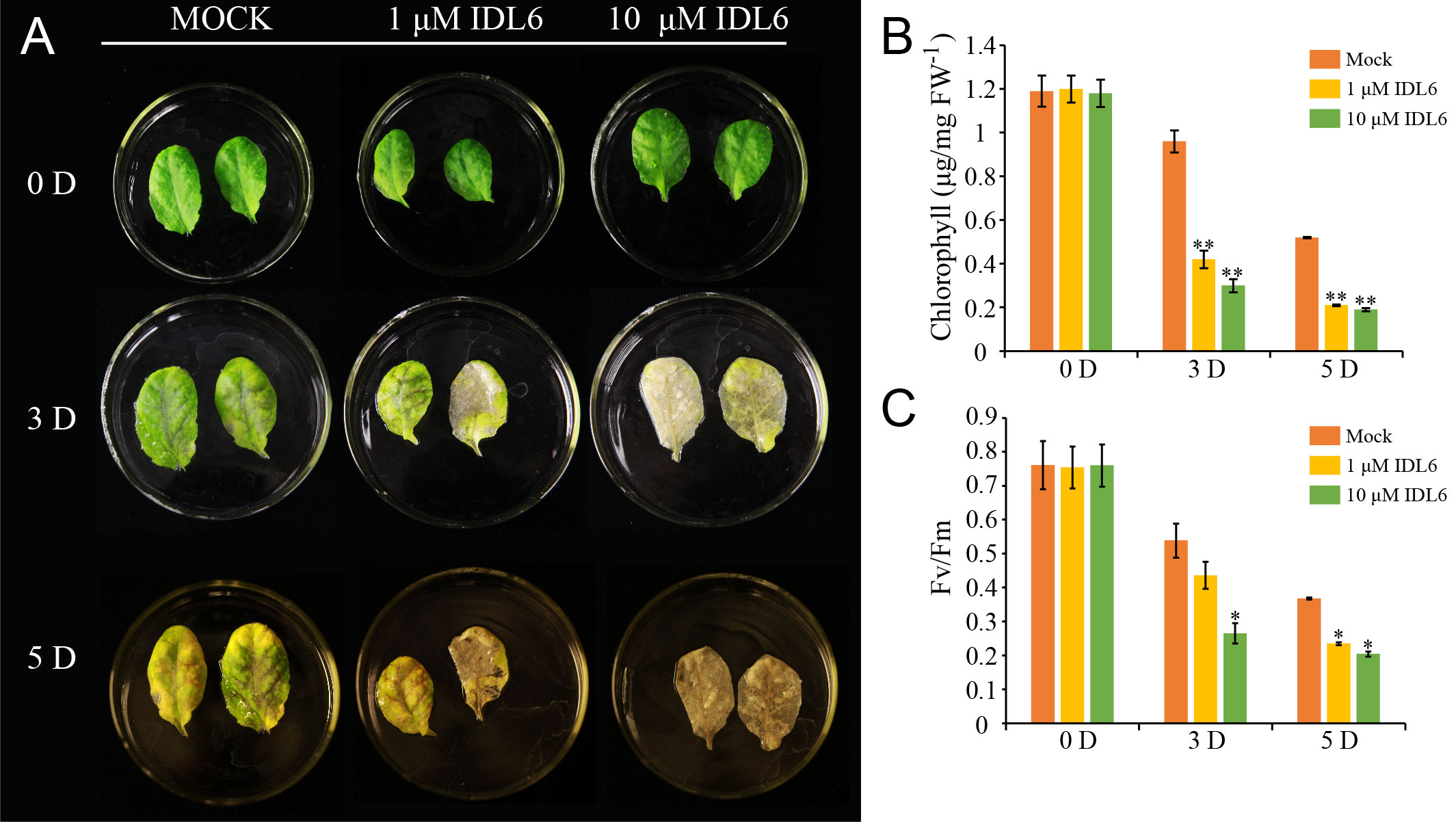

Supplement: Supplementary Figure 1 — Sequence information of the IDL6 peptide. [file Data_Sheet_1.ZIP › Supplementary Material/Supplementary Figure 5.jpg]
